# A role for activatory KIR/HLA complexes in HIV-associated Kaposi’s sarcoma development

**DOI:** 10.1186/1742-4690-8-S1-A209

**Published:** 2011-06-06

**Authors:** Simone Agostini, Franca R Guerini, Alessandra Bandera, Roberta Mancuso, Ambra Hernis, Cristina Agliardi, Francesca Sabbatici, Ilaria Beretta, Caterina Fusco, Andrea Gori, Mario Clerici

**Affiliations:** 1 Don Gnocchi Foundation Onlus, Milan, 20148, Italy; 2 Dept. internal medicine, University of Milan-Bicocca, Milan, 20148, Italy; 3 Immunoematology and transfusional unit, Hosp. Cardarelli, Napoli, Italy; 4 Dept. biomedical sciences and technologies, University of Milan, Milan, 20148, Italy

## 

Kaposi’s Sarcoma is a vascular tumour caused by the Kaposi’s sarcoma-associated herpesvirus (KSHV). Although KSHV and HIV co-infection is often observed in HIV disease, the majority of patients nevertheless do not develop KS. Innate immunity, and in particular human leukocyte antigen alleles (HLA) and killer cell immunoglobulin-like receptor (KIR) are suspected to play a role in the development of viral associated carcinomas. We verified possible correlations between KIR/HLA complexes and KS by analyzing 82 HIV subjects: 15 with KS (KSpos), 32 KSHV-infected without KS (KSHVpos/KSneg) and 35 KSHV-uninfected (KSHVneg/KSneg); results were compared to those of 103 age-and sex-matched KHSV-seronegative individuals (HC).

Molecular genotyped was performed by Single Specific Primer (SSP) method for HLA class I, and KIR alleles. Chi-square analysis Yates corrected (py) and Fisher exact test (pf) were evaluated when appropriated. Results showed a statistically higher frequency of the activating KIR2DS2 allele in KSpos subjects (93.3%) than either in KSHVpos/KSneg (46.8%, py=0.02), or KSHVneg/KSneg (51.4%,py=0.008) patients, and HC (42.7%, py=0.02). Moreover, the homozygous genotype KIR2DS2+/2DL2- was present in 3/15 KSpos, but in none KSHVpos/KSneg (pf=0.03) or KSHVneg/KSneg (pf=0.02) and only in 1/103 HC (pf=0.006).

Finally there was an higher frequency of KIR2DS2/HLAC-1 functional complex in KSpos (66.7%) than in all the other groups (40.6%, 40.0%, 43.7%, respectively), though not statistically significant.

Results herein, although stemming from analyses performed on a small number of individuals, suggest that HIV and KSHV co-infected subjects in whom activating KIRs and activating KIR-HLA ligands functional complexes are detected, are more prone to develop KS.

